# The Aseptic and Antiseptic Treatment of Wounds

**Published:** 1894-12

**Authors:** William B. Crawford

**Affiliations:** Augusta, Ga.


### THE ASEPTIC AND ANTISEPTIC TREATMENT OF WOUNDS.*

By WILLIAM B. CRAWFORD, M.D.,

Augusta, Ga.

During the past fifteen years, perhaps no subject has furnished a theme for more frequent discussion, in societies of medicine and in medical journals, than the one which forms the title of this paper.

Definite forms of action have been formulated and agreed upon, but there has been, and is still, quite a diversity of opinion in the profession as to the most feasible methods to be adopted, how far these methods should be carried on and when they should cease.

In entering upon any scientific investigation, the investigator should enter the field free from any prejudice, and prepared to accept facts as they are rather than bend the facts to any preconceived ideas he may have upon the subject he is investigating. I have aimed at following out this liberal line of thought, and in the following paragraphs it will be my object to set forth my own views upon asepsis, and antisepsis as applied to the treatment of wounds; at the same time I invite and shall expect the most searching criticism from the members of this academy.

Before entering into a consideration of the purely scientific or surgical aspect of my subject, it may be well to briefly contrast the surgical teachings of former times with the views held to-day. And by this I do not mean that I shall consume your time with a lengthy review of the history of surgery for the past century; it shall rather be my purpose to set forth as concisely as possible the great progress which this branch of medicine has made of late, very much of which advancement is to be attributed to aseptic and antiseptic methods, which, however, yet in their infancy have come to stay, and give every promise of greater possibilities for the surgery of the future.

In scanning the pages of a recent issue of a medical journal, I found the following statement: "A hundred years or so ago, our

*Read before the Augusta Academy of Medicine, October 3, 1894.

ranks were composed mainly of quacks and of impractical dreamers." Recognizing the important advancements in medical science, and remembering that humility is characteristic of true knowledge, I must say that I do not approve of this disparagement of our professional ancestors, nor of exalting too highly the attainments of the profession of to-day.

If the names upon the roll of medical teachers of a hundred years or so age be compared with the names upon the roll of medical teachers of to-day, it will be found that there were quite as intelligent and able men then as now, and such a comparison will do no discredit to the men of the last century. But that we of to-day are in advance of them in possessing a more perfect knowledge of etiology and pathology, which knowledge enables us to arrive more nearly at precision in treatment, is a fact that cannot be denied. And in no branch of medical science has this knowledge marked so great improvements as in surgery. As furnishing an idea of the methods employed in the treatment of wounds during the last century, allow me to quote the following extracts from the "Discourses on the Nature and Cure of wounds," published at Edinburgh in 1795, by John Bell, the eminent Scottish surgeon and anatomist. He says:

"Thirty years ago surgeons had no settled notions that cut surfaces might be made to adhere; they had no motive for saving the skin, or when they had saved it, they had no idea how it should be used nor how much it might contribute to a speedy cure; if they extirpated a tumor, they cut away along with it all the surrounding skin; if they performed the trepan, they performed in a most regular manner the preliminary operation which they chose to call scalping; or in plain terms they cut away six or eight inches of the skin which should have saved the fractured skull from exfoliation, and should have immediately covered and defended the brain. In performing amputation, they cut by one stroke down to the bone; and even when they performed the flap amputation, they dressed their stump and flap as distinct sores. An exfoliation of the bone in those older operations was a thing unavoidable; so that it was a part of their art and skill to procure exfoliation."

When the French surgeons had declared that not only did their flap amputation procure an easy and perfect cure, but affirmed that often in three days the flesh of such a stump had adhered, O'Halleran, a contemporary of Bell's, and whom Bell characterizes as an excellent and most judicious surgeon, whose doctrine and practice was followed by all the best surgeons of that day, made the following reply :

"I would ask the most ignorant tyro in our profession, whether he ever saw or heard even of a wound, though no more than an inch long, united in so short a time?" adding, "These tales are told with more confidence than veracity; healing by inosculation, by the first intention, by immediate coalescence without suppuration, is merely chimerical and opposite to the rules of nature."

Other similar quotations might be made from this interesting book, but this suffices to show that our ancestors were totally ignorant of much that is perfectly familiar to us. And who can tell but that the contrast between the profession as it is now, and as it will be a hundred years hence, will be greater than is that between the former and the profession as it was a hundred years ago?

The history of the influence of bacteriological studies, in the development of aseptic and antiseptic surgery is one of the most interesting and important of the modern records in the science of medicine. The wildest dreams of our imagination could have never foretold the momentous consequences that would result from the discovery and investigations of bacteria and other minute micro-organisms. It is a striking illustration of the fact that we often do not, and cannot appreciate how far-reaching and important a scientific discovery may be, even when at the time it seems to have no practical use or benefit. It is chiefly to the labors of the great Professor Pasteur that we owe the foundation upon which aseptic and antiseptic surgery has been built. He demonstrated that the processes of fermentation and putrefaction are entirely due to the presence and action of these microscopic germs, and if these are absent such changes will never take place. Professor Tyndall, of England, followed this and proved conclusively that if we would completely exclude the living germs of bacteria of the air from infusions of animal or vegetable matter, they could be kept indefinitely. Infusions, such as beef-tea, mutton-broth, chickenbroth, and infusions of hay and other vegetable matters may be kept for years, if after boiling to sterilize or kill the living germs contained in them, they are hermetically sealed to exclude the air which contains the germs. He also found that if the mouths of vessels containing these infusions are plugged up with antiseptic cotton to filter out the germs as the air passes in and out, these infusions could be preserved indefinitely.

To Professor Lister we owe the grand idea of excluding the bacteria and other germs from wounds, thus creating the then new science of antiseptic surgery. The practice of surgery has been almost revolutionized by the teachings of Lister, though it is perfectly true that antiseptic surgery as now practiced is very different from, and far superior to, the cumbrous procedures and dressings first devised and practiced by the father of antiseptic surgery; nevetheless, it remains, and ever will remain a fact, that no man has done more to improve the methods and to bring success to the surgical art, than this great apostle of modern cleanliness.

In operative surgery, aseptic and antiseptic measures form the very corner-stone of success. The technique of these measures, as applied to operative surgery, is too well understood for me to occupy your time in the recital of it. I will say, however, that on the principle that "an ounce of prevention is worth a pound of cure," aseptic methods are here to be commended in preference to antiseptic methods. If we hold strictly to asepsis, then we will find no need for antisepsis. And how are we to maintain thorough asepsis in operative proceedings?

1st. We must have a clean field. 2d. We must have clean instruments and dressings. 3d. The surgeon and his assistants must be clean.

Under the head of a clean field, I need say no more than insist upon a clean operating-room, with absolute cleanliness of all the surroundings, and proper ventilation, though it is unfortunately too true, that outside of hospital practice, we do not often find it possible to have a perfectly clean field for operating work.

As to cleanliness of instruments and surgical appliances, I suggest that they be sterilized by first boiling in water containing soda, and then being exposed to dry heat above the temperature of boiling water for at least one-half hour. For dressings, the many layers of protective gauze, mackintosh, etc., as formerly practiced, have been abolished, and are now replaced by a simple layer of iodoform gauze, with an abundant layer of sterilized cotton, firmly retained by bandages. Concerning the commecrial dressings, as ordinarily used, I shall have something to say in a succeeding paragraph.

As to cleanliness of the surgeon and his assistants, I will relate what a prominent professor of surgery in this country told me of an experience he had in Europe some years ago.

He was invited to assist in an operation in one of the hospitals in Paris, but before being admitted into the operating-room, was asked if he had taken a bath, and invested himself with clean underwear. Upon giving a negative answer, he was requested to do so, and the operation was delayed until he performed these precautionary measures. Since that time, however, it has been found that these extreme precautions are no longer necessary. But too much stress cannot be laid upon cleanliness of the hands and especially of the nails, when we remember that bacteriologists tell us that we may carry thousands of germs under the finger nails. In operative proceedings care should be taken that the parts operated upon be given as much rest as possible, that the circulation be not obstructed by drawing the sutures too tight, and in the choice of needles and sutures, select the smallest size possible to meet the requirements of the case, because by so doing less opportunity is afforded for the lodgment of germs.

As an evidence that asepsis is preferable to antisepsis in operative surgery, it is interesting to note that the antiseptic sprays, once so universally applied, are now being abandoned because of the growing disbelief in the ordinary antiseptic solutions when used as germicides. A solution of carbolic acid has been shown to be a very weak germicide, and the same may be said of a solution of boric acid, and of the other solutions used commonly for this purpose. Bichloride of mercury has until the present time been our sheet anchor as a germicide, but recent investigations have shown that solutions of mercuric chloride are often inert as a germicide. I would say of mercuric chloride as of carbolic acid, that they are both to be feared because of their toxic properties. It is doubtful if they do not often irritate the tissues, and sometimes may be the cause of very serious results.

The consensus of opinion of the masters of surgery is fast settling upon the conviction that there are practically but two methods for keeping wounds aseptic. One is to keep wounds made during surgical operations as dry as possible, and the other is to use only recently boiled water in contact with them.

Aseptic surgery, as practiced in the great hospitals of the world to-day, is carried out with such a perfection of detail, and with such successful results as was never dreamed of by our professional brethren of a generation or so ago.

And the methods of operating are of the simplest character. The arms and hands of the operator and his assistants are thoroughly cleansed by scrubbing for five minutes with soap and water, then subjected to a 1–2000 solution of bichloride of mercury, after which they are rinsed in alcohol. That part of the patient's body, where the operation is to be performed, is also made thoroughly clean by shaving of hair, if necessary, and then subjected to thorough scrubbing.

As few instruments as possible are used, and these have recently been sterilized by the method already described. No sponges are used. Their places being supplied by balls of sterilized cotton or gauze. In cases of amputation, no ligatures are used for the vessels except such as silk for the larger vessels, or catgut for the smaller ones, and these are cut short. No drainage-tubes are used, nor in fact, anything that will prevent union by the first intention. No plasters are used on the stump as formerly to keep the flaps in close apposition, because the use of adhesive plasters does not belong to aseptic surgery. The stump is dusted with iodoform, and iodoform gauze is applied, then a very liberal supply of sterilized cotton-wool firmly retained by properly applied bandages. The operator must not, of course, touch anything from which infection might be carried to the wound. He is not even allowed to pass his hand over his own face, or to touch any part of his body with his hands during an operation. It is a remarkable fact that almost all the histories of cases operated upon under these precautions, show recovery without rise of temperature or suppuration.

Among the various wounds resulting from accident which we are called upon to treat, the lacerated and contused wounds constitute a very large proportion. Before laying down certain principles governing the treatment of such wounds, let us first inquire into their nature and pathology.

The initial injury varies from the slightest irritation to a complete destruction of vitality, so that the process of repair varies from what might be termed a process of physiological regeneration to the gravest termination of inflammation, suppuration, gangrene, or mortification. I would say then, that in order to treat such wounds intelligently, we should be perfectly familiar with the pathology of the inflammatory process. We must appreciate the minute changes going on within the vessel walls; we must remember the tendency to accumulation of blood cells, serum, new formed connective cells, in the tissues surrounding blood-vessels; we must realize the tendency to stasis, the blocking up of the over-worked lymphatics, the obstruction to circulation, the shutting off of nutrition with consequent death; and above all we must remember, that even when mortification does occur, that it can be limited, or controlled if we keep from it the pathogenic microbe.

It may be safely said that every lacerated wound which is allowed to pursue its course without primary cleansing, and subsequent avoidance of dirt or filth, will suppurate; therefore, the prophylactic treatment of inflammation is the first indication to be met, and that is nothing more nor less than absolute cleanliness, asepsis. In this class of injuries there is always an accompaniment of dirt which must be at once attended to. No matter how small the wound, the scrub brush, with plenty of soap and water, must be used; for a very small injury filled with dirt and grease may be the starting point of an inflammation that will result in the loss of a limb or even the life of the patient.

The primary cleansing of a lacerated wound is of such importance that it should always be attended to, even should it be so painful as to require the use of an anesthetic for the process. Cleanse it with soap and brush and a 1–2000 solution of bichloride of mercury; then remember that however thorough has been our cleansing, it is barely possible that some septic matter might still be left in the wound, so that we cannot, as in incised wounds, rely entirely upon sterilized dressings, but must use antiseptic dressings. And since antiseptics are more active when moist, and since the devitalized tissues are greatly benefited by warmth, it is highly probable that these injuries, or a majority of them, are best handled by dressing them in the first instance with warm, moist bichloride gauze. It sometimes happens that in spite of our precautions suppuration does occur, in which case the use of peroxide of hydrogen, with proper drainage, will prove very effective treatment. In cases of wounds which have been exposed for some time, and which have already become ill-smelling, suppurating, and inflamed, I have seen most favorable results follow the use of peroxide of hydrogen, after all the wound recesses and cavities have been laid open as far as possible to admit of free drainage. The consecutive cleansing of lacerated wounds, to admit of the free escape of wound débris of every kind during the period of repair, will require the fullest provisions for drainage from all its recesses. For this reason a too zealous use of sutures is to be deprecated. It is very true that when the lacerated wound can be treated so as to be transformed into a clean, incised wound, sutures are to be used the same as for incised wounds. A few stititches may also be employed if the wound be large, and there be much tendency to gaping; but, as a rule, they are contraindicated, it being best to rely chiefly upon the support of judicious bandaging with the use of the moist bichloride rollers. Adhesive plasters should never be used in the treatment of lacerated wounds, because of the great danger of septic infection, which arises from their use. Indeed, I doubt very much if the use of the adhesive plaster rightly belongs anywhere in surgical practice. In the commercial articles commonly used there is no pretense, and certainly no fact of asepticity. All sorts of animal refuse are doubtless made use of in their manufacture; old animal skins, horns, hoofs, parings, etc., and how filthy the process of concoction and preparation may have been, of course no one is capable of saying.

In the local treatment of lacerated wounds, there is often an indication for heat and moisture, because the devitalized tissues are greatly benefited by warmth and moisture. In order to supply these conditions it has been a common practice to employ poultices, but many of the leading surgeons have discarded the use of poultices as not being in line with thorough aseptic practice, and are using in their stead hot bichloride fomentations with most satisfactory results.

The class of wounds known as contused punctured wounds, such as those made by the thrusting into the tissues of a splinter of wood, a nail, a bayonet, or other substance which is capable of making a deep and narrow wound track, present some difficulty in treatment, by reason of the trouble they may give in securing their disinfection and drainage. The first indication in the treatment of such wounds, after removing the puncturing substance, is to cleanse and disinfect those parts of the wound that are accessible. If speedy repair without disturbance does not take place, but there develops an inflammation of the deeper portions of the wound, then should free incisions be made for the relief of the pent-up effusions, and an antiseptic solution be applied. And for such a wound carbolic acid is preferable, because of its volatility and diffusibility. Use a five per cent. solution until the wound has been rendered completely aseptic, though during subsequent care of the wound a more dilute solution may be used. Throughout the future course of such a wound, greater care and watchfulness against the possible redevelopment of septicity will be required than would be deemed necessary in the management of a wound aseptic from the first.

Gunshot wounds, in whatever way inflicted, partake of the nature of contused and punctured wounds, and the same general principles of treatment should be applied as to the contused and punctured varieties. The disastrous results of gunshot wounds, which are not quickly fatal, are consequent upon inflammatory, suppurative, and infective disturbances which complicate their repair. These disturbances have their origin in septic contamination of the wound track, and gain their aggravated proportions from the natural obstacles which the wound track presents to the escape of the septic matters afforded by the contaminated wound secretions and débris. The prevention of septic contamination of the wound track, then, is to be considered of first importance. Immediate antiseptic occlusion, or the application to the external wound of tampons of absorbent antiseptic material, as early as possible after the infliction of the wound, should be done, for no period of time can safely be allowed to lapse between the reception of the injury and its protection from septic invasion. Absorbent cotton impregnated with iodoform or boracic acid may be selected for such occlusive purposes. The tampon is to be applied directly to the wound, and then oiled paper used as an external covering, the whole to be secured with a bandage. The chief obstacle to the general adoption of the practice of primarily sealing up the aperture of a gunshot wound lies in the importance which has generally been attached to the early removal of the missile when imbedded. We are told, however, by such eminent authority as Esmarch, that "the damage done by the bullet is caused by it in its course; the harm that is added comes mostly from the examiner's fingers." The mere lodgment of a bullet, therefore, in the tissues is not an indication for opening up the wound track by an exploring finger or probe, and exposing the wound to the septic contamination which such a maneuvre would entail. It is not justifiable to disturb the wound until distinct evidence has appeared that the missile, or some foreign body with it, as for instance a patch of the clothing, is seriously disturbing the repair of the wound by its presence.

And now a word as to surgical dressings:

In view of the attention bestowed by modern surgeons upon the details of the antiseptic method of wound treatment, the present style of surgical dressings has reached a high order of excellence. But while our pharmacopeia provides the pharmacist with an official guide for the manufacture of various preparations for the physician's use, such as fluid extracts and tinctures, for instance, it seems strange that no effort has been made to adopt a uniform standard for the preparation of surgical dressings, or the various articles belonging to what may be termed the surgical materia medica, such as medicated gauzes, suture materials, cottons, etc. Under existing conditions, the surgeon is compelled to rely upon the assurances of the manufacturer that the dressings ordered have been scientifically prepared, and contain the prescribed quantity of the medicament.

This circumstance had been taken advantage of by unscrupulous manufacturers, to offer to the profession goods of an inferior quality, especially if the medicament employed is expensive, as in the case of iodoform gauze. The undiscriminating buyer is persuaded that he has obtained the genuine article because the gauze has the characteristic odor and color of iodoform. He loses sight of the fact that the gauze may be stained yellow in other ways, and that a very little iodoform goes a great ways as regards odor. True, these evils can be obviated if every surgeon will prepare his own dressings, but this is a laborious proceeding, which is unnecessary if we understand what we want, and will take nothing else. Even the honest maker is hampered in his efforts to produce a perfect article, by the lack of interest in the subject by many surgeons, and his inability to learn their exact wants. Let the profession institute a definite standard in the preparation of surgical dressings; let the requirements of the consumers become more exacting, and the makers will raise the standard of their products accordingly.

Let there be as definite plans mapped out for the preparation of these dressings as for the preparation of fluid extracts and tinctures of the pharmacopeia, and the day will soon dawn when reliable, scientifically prepared dressings will be the rule rather than the exception.

---

### DIARRHEA OF DENTITION.

In the *Ciencias Medicas, de Barcelona* in the treatment of the diarrhea of dentition, the following preparation is found of use:

℞ Subnitrate of bismuth .................... 4 gms (℥ j).

Lime water .................... (℥ jj).

Fennel water .................... 100 " (℥ iijss).

Syr. orange peel .................... 20 " (℥ v).

One teaspoonful every three hours.

—*Med. and Surg. Rep.*

---

### FOR THE GREEN DIARRHEA OF INFANTS.

℞ Acidi lactici diluti .................... *m* iv.

Tincturæ limonis .................... *m* i.

Syrupi,

Aquæ, of each .................... ℥ ii.

M. A teaspoonful thrice daily after suckling.—*Practitioner*.

